# The Epithelial-Mesenchymal Transition Initiated by Malignant Ascites Underlies the Transmesothelial Invasion of Ovarian Cancer Cells

**DOI:** 10.3390/ijms20010137

**Published:** 2019-01-02

**Authors:** Martyna Pakuła, Justyna Mikuła-Pietrasik, Anna Witucka, Katarzyna Kostka-Jeziorny, Paweł Uruski, Rafał Moszyński, Eryk Naumowicz, Stefan Sajdak, Andrzej Tykarski, Krzysztof Książek

**Affiliations:** 1 Department of Hypertensiology, Angiology and Internal Medicine, Poznań University of Medical Sciences, Długa 1/2 Str., 61-848 Poznań, Poland; mpakula@ump.edu.pl (M.P.); jmikula@ump.edu.pl (J.M.-P.); a.witucka@ump.edu.pl (A.W.); kostkajeziorny@ump.edu.pl (K.K.-J.); puruski@ump.edu.pl (P.U.); tykarski@o2.pl (A.T.); 2 Division of Gynecological Surgery, Poznań University of Medical Sciences, Polna 33 Str., 60-535 Poznań, Poland; rafalmoszynski@gmail.com (R.M.); ssajdak@ump.edu.pl (S.S.); 3 General Surgery Ward, Medical Centre HCP, 28 czerwca 1956 r. 223/229 Str., 61-485 Poznań, Poland; eryknaumowicz777@gmail.com

**Keywords:** epithelial-mesenchymal transition, malignant ascites, ovarian cancer

## Abstract

The role of the epithelial-mesenchymal transition (EMT) in ovarian cancer cell progression is unquestioned. In this report, we describe that malignant ascites, fluid that accumulates in the peritoneal cavity in a large group of patients with ovarian cancer, stimulate EMT in two representative ovarian cancer cell lines (A2780, SKOV-3). In addition, we identify the ascites-derived mediators of EMT and signaling pathways initiated in the cancer cells that underlie this phenomenon. Finally, we demonstrate that EMT induced in the cancer cells in response to the malignant ascites contributes to their increased transmesothelial invasion. Altogether, our study provides new insight into the mechanistic aspects of the malignant ascites-dependent exacerbation of the intraperitoneal progression of ovarian cancer.

## 1. Introduction

Ovarian cancer is the most lethal gynecologic malignancy, whose pathophysiology is still extensively studied due to numerous challenges arising from the unique heterogeneity of the disease [1]. One of the most intriguing aspects of ovarian cancer progression is the formation of intraperitoneal metastases. Recent years have provided evidence that this process requires a permissive microenvironment within the peritoneal cavity, created to a large extent by the presence and activity of malignant ascites. This term refers to pathologic fluid that accumulates in the abdomen in a group of patients, mostly in the advanced stages of the disease [2]. Malignant ascites have been found to create hospitable conditions for tumor expansion by suppressing peritoneal inflammatory reactions [3], inducing angiogenesis [4], and promoting cancer cell proliferation [5] and migration [6].

Recently, we discovered that malignant ascites modulate the efficacy of the transmesothelial invasion of ovarian cancer cells. This effect was linked to their ability to decrease the expression of several junctional proteins (connexin 43, E-cadherin, occludin, and desmoglein) in peritoneal mesothelial cells (PMCs), in a p38 MAPK- and NF-κB-dependent manner [7]. Increased invasive properties of ovarian cancer cells exposed to the ascites were also revealed by other authors, who additionally showed that the fluid may induce the formation of cellular spheroids [8]. Intriguingly, ovarian cancer spheroids isolated from the malignant ascites have been found to display features of the epithelial-mesenchymal transition (EMT), a process in which cancer cells lose their epithelial characteristics and transform into far more aggressive spindle-shaped cells [9].

The observations mentioned above prompted us to continue our research on the ascites-related transmesothelial invasiveness of ovarian cancer cells. In this project, we examined whether the malignant ascites induce EMT in ovarian cancer cells, and, if so, what is/are the trigger(s) of this phenomenon in the fluid and which signaling pathways are engaged in the initiation of this process in cancer cells. 

## 2. Results

### 2.1. Malignant Ascites Induce EMT in Ovarian Cancer Cells

Two ovarian cancer cell lines, A2780 and SKOV-3, were exposed to standard growth medium and benign and malignant ascites. Next, two representative markers of EMT-related cellular rearrangement, E-cadherin and vimentin, were examined. Quantitative immunofluorescence-based analysis revealed that the expression of both proteins in cells treated with the benign fluid did not alter from that characterizing cells maintained under the optimal culture conditions. 

**Figure 1:**
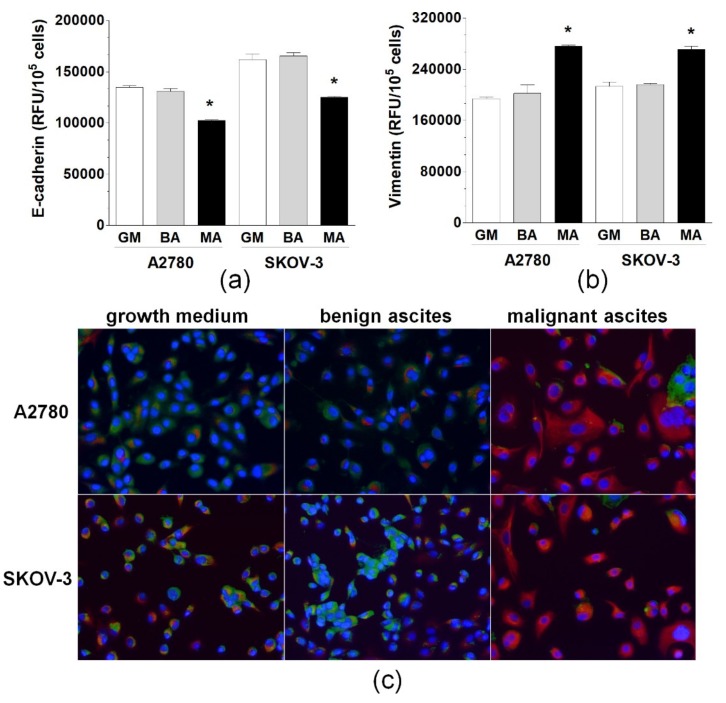
Expression of E-cadherin (**a**) and vimentin (**b**) in A2780 and SKOV-3 cells exposed to growth medium (GM), benign ascites (BA), and malignant ascites (MA). Panel (**c**) shows representative results of the dual immunostaining of E-cadherin (green) and vimentin (red) in the cancer cells. DAPI staining (blue) indicates the nuclei (magnification ×100). Asterisks (*) indicate significant differences (*p* < 0.05) compared with ovarian cancer cells exposed to benign ascites. Experiments were performed with the cancer cells used in hexaplicates. The benign and the malignant ascites were obtained from eight different patients per group. RFU—relative fluorescence units.

**Figure 2:**
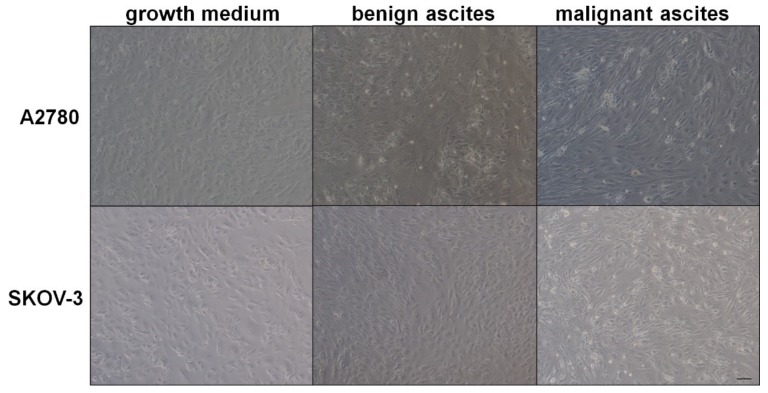
Representative pictures showing the development of the spindle-shaped morphology typical for cells undergoing epithelial-mesenchymal transition (EMT) in ovarian cancer cells subjected to malignant ascites (magnification ×50; bar 100 μm).

Conversely, when the cells were exposed to the malignant fluid, the expression of E-cadherin was decreased, whereas the expression of vimentin was increased significantly in both types of cancer cells. These changes were reflected microscopically using dual immunofluorescence staining of E-cadherin and vimentin (Figure 1). Moreover, the development of EMT in ovarian cancer cells treated with malignant ascites was confirmed microscopically in a brightfield, according to specific, morphological cell reorganization (Figure 2).

### 2.2. Malignant Ascites Are Rich in Proteins That Induce EMT in Ovarian Cancer Cells

**Figure 3:**
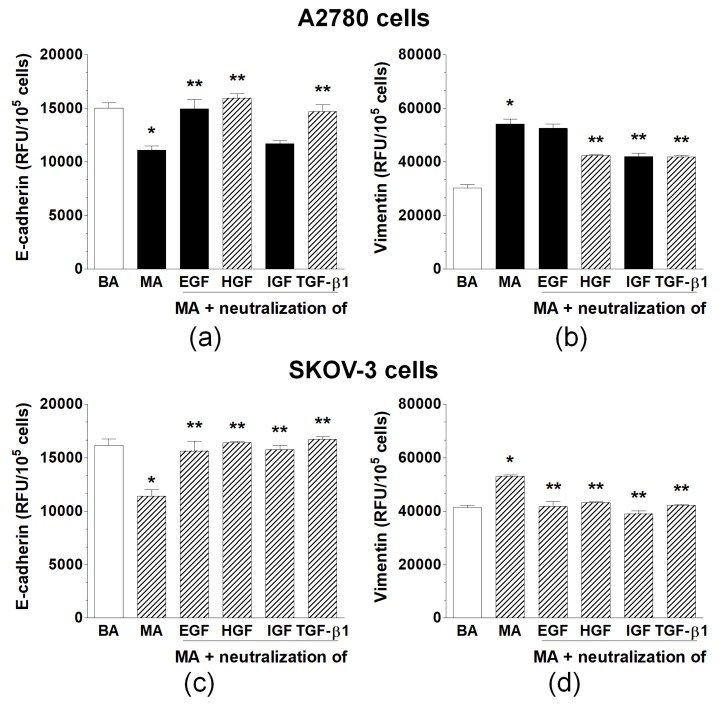
Expression of E-cadherin and vimentin in A2780 (**a**,**b**) and SKOV-3 cells (**c**,**d**) exposed to benign ascites (BA), malignant ascites (MA), and MA pre-incubated with specific antibodies against EGF, HGF, IGF, and TGF-β1. Hatched bars indicate the mediators whose neutralization consistently reversed the MA-dependent changes in the level of E-cadherin or vimentin. Single asterisks (*) indicate significant differences (*p* < 0.05) compared with ovarian cancer cells exposed to benign ascites. Double asterisks (**) indicate significant differences (*p* < 0.05) compared with the cells exposed to MA. Experiments were performed with the cancer cells used in hexaplicates. The benign and malignant ascites were obtained from eight different patients per group. RFU—relative fluorescence units.

Specific neutralizing antibodies directed against four agents known from the literature to cause EMT—that is, EGF, HGF, IGF-1, and TGF-β1—were added to the malignant ascites to establish whether some of these agents may be responsible for EMT development in ovarian cancer cells. Significantly, only these antibodies who inhibited EMT-associated changes in both E-cadherin and vimentin expression were considered as indicating the plausible mediator(s) of EMT. The analysis performed in accordance with this regimen revealed that the development of EMT in A2780 cells was elicited by HGF and TGF-β1. However, in SKOV-3 cells, EMT was triggered by EGF, HGF, IGF-1, and TGF-β1 (Figure 3).

### 2.3. Induction of EMT in Ovarian Cancer Cells Exposed to Malignant Ascites Is a Multi-Signaling Phenomenon

**Figure 4:**
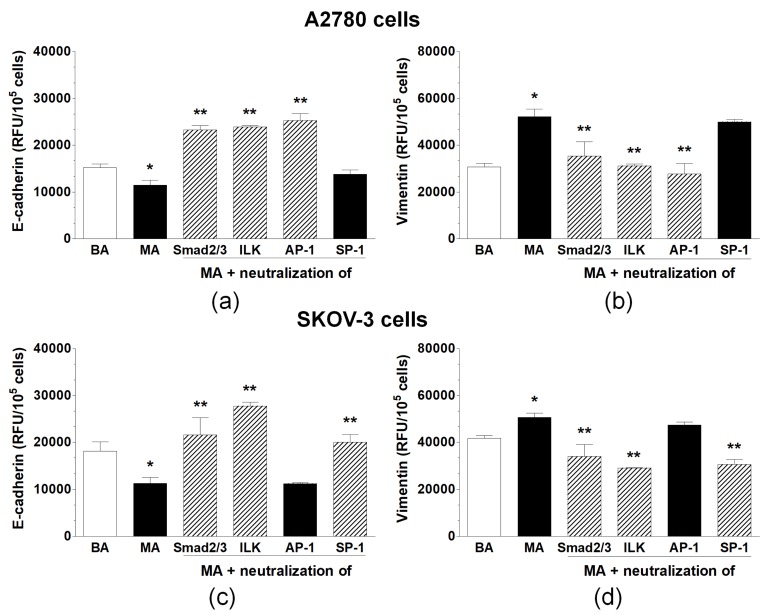
Expression of E-cadherin and vimentin in A2780 (**a**,**b**) and SKOV-3 cells (**c**,**d**) exposed to benign ascites (BA), malignant ascites (MA), and MA following cancer cell pre-incubation with the inhibitors of Smad 2/3, ILK, AP-1, and SP-1. Hatched bars indicate these signaling molecules whose blockade consistently reversed the MA-dependent changes in either the E-cadherin or vimentin level. Single asterisks (*) indicate significant differences (*p* < 0.05) compared with ovarian cancer cells exposed to benign ascites. Double asterisks (**) indicate significant differences (*p* < 0.05) compared with the cells exposed to MA. Experiments were performed with the cancer cells used in hexaplicates. The benign and malignant ascites were obtained from eight different patients per group. RFU—relative fluorescence units.

Specific chemical inhibitors against four molecules linked with EMT—that is, Smad 2/3, ILK, AP-1, and SP-1—were added to ovarian cancer cells prior to their exposure to the malignant ascites to identify the signaling pathway(s) responsible for the induction of this process. Similarly, as in the case of soluble mediators, only these inhibitors that consistently blocked EMT-related changes in E-cadherin and vimentin were considered to indicate plausible pathways involved in EMT development. In A2780 cells, it was shown that the alterations in the expression levels of both tested proteins were inhibited upon the blockade of Smad 2/3, ILK, and AP-1. However, in SKOV-3 cells, the same effect was reached by the inhibition of Smad 2/3, ILK, and SP-1 (Figure 4).

### 2.4. Transmesothelial Invasion of Ovarian Cancer Cells Promoted by Malignant Ascites Is Inhibited by Interference with the Mediators and Signaling Pathways Engaged in EMT Development 

**Figure 5:**
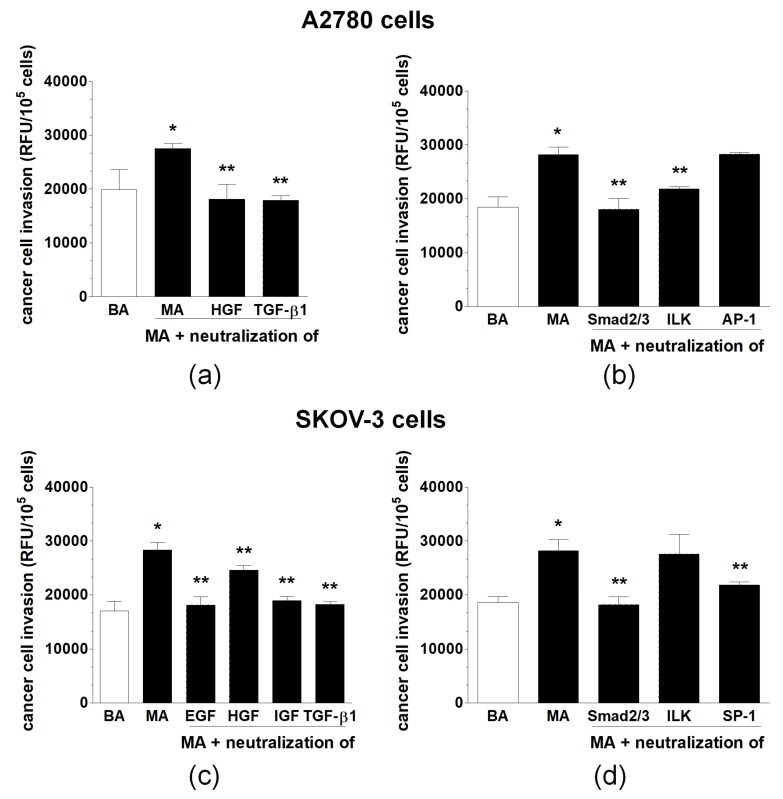
Invasion of A2780 and SKOV-3 cells through monolayered peritoneal mesothelial cells upon preincubation of malignant ascites (serving as the chemoattractant) with the antibodies neutralizing possible mediators of EMT (**a**,**c**) and following preincubation of the cancer cells with the inhibitors neutralizing EMT-associated signaling molecules (**b**,**d**). Single asterisks (*) indicate significant differences (*p* < 0.05) compared with ovarian cancer cells exposed to benign ascites. Double asterisks (**) indicate significant differences (*p* < 0.05) compared with the cells exposed to MA. Experiments were performed with cancer cells used in hexaplicates. The benign and malignant ascites were obtained from eight different patients per group. RFU—relative fluorescence units.

Invasion of ovarian cancer cells across monolayered PMCs was used to compare the effects of benign and malignant ascites, as well as to verify whether the inhibition of the identified EMT-related mediators and signaling pathways will attenuate this phenomenon. Direct comparison of the invasive properties of A2780 and SKOV-3 cells pre-exposed to both types of fluids showed that the motility of both cancer lines exposed to malignant ascites was considerably higher. When the process was examined using cancer cells treated with the malignant ascites preincubated with the antibodies neutralizing the mediators of EMT, the invasiveness of A2780 cells was significantly reduced upon the neutralization of HGF and TGF-β1. The invasion of SKOV-3 cells was efficiently restricted upon the inhibition of EGF, IGF, HGF, and TGF-β1. Regarding signaling pathways modulating cancer cell invasion, the reduction of A2780 cell motility was observed upon the blockade of Smad 2/3 and ILK but not AP-1. In the case of SKOV-3 cells, their invasion was decreased upon the inhibition of Smad 2/3 and SP-1 but not ILK (Figure 5). 

## 3. Discussion

An increasing body of evidence has indicated that malignant ascites may determine the high invasiveness of ovarian cancer cells, plausibly via the induction of the EMT phenomenon [9]. At the same time, the mechanistic aspects of this process have been poorly explored. This situation is additionally blurred by ovarian cancer cells being very heterogeneous and their attack on the peritoneal cavity not always being associated with EMT development [10]. 

In this study, we found that malignant ascites generated by serous ovarian tumors induce EMT in two representative ovarian cancer cell lines (A2780, SKOV-3), supporting the opinion regarding the procancerous nature of this fluid [11]. This statement is even more evident considering the lack of EMT in cancer cells treated with benign fluids or standard growth medium. Indeed, the composition of malignant and benign ascites differs significantly, corresponding to distinct behaviors of cancer cells exposed to these fluids [5]. 

An initiated EMT may explain the increased effectiveness of the transmesothelial invasion of ovarian cancer cells and may be considered as an additional pathomechanism of this process, next to that associated with the decreased expression of junctional proteins in the peritoneal mesothelium [7]. Recently, a similar shift toward the mesenchymal ovarian cancer cell phenotype after their exposure to the malignant ascites was reported as favoring their proliferation and migration. In that study, a redeployment of αV integrins into cells was observed and was attributed to the establishment of the invasive, EMT-related behavior of the cancer cells [8].

To join a debate regarding the mechanism of the ascites-dependent EMT, we arbitrarily selected four proteins that are known to induce the process in various cell types (EGF [12], IGF-1 [13], HGF [14], TGF-β1 [15]) and four signaling molecules (Smad 2/3 [16], ILK [17], AP-1 [18], SP-1 [19]) linked with this phenomenon. Regarding the former group, some differences were established in the two cancer cell lines tested. The effect in A2780 cells appeared to be related to the activity of HGF and TGF-β1. However, in SKOV-3 cells, all four agents were involved. Regarding the signaling routes, the situation was similar. EMT in A2780 was associated with the activity of Smad 2/3, ILK, and SP-1. However, in SKOV-3 cells, the activation of Smad 2/3, ILK and AP-1 was critical. The differences in the mechanisms underlying EMT in the two analyzed types of ovarian cancer cells confirmed the heterogeneity of the disease and support previous reports postulating broad molecular and genetic differences between various ovarian cancer cell types, even of the same histological origin. It should be mentioned, at the moment, that both cell lines used in the study do not resemble high-grade serous ovarian cancer (HGSOC), as they do not have the typical copy number and TP53 mutation profiles found in HGSOC [20].

Nonetheless, our findings extend the view on the pathomechanism of ascites-induced EMT. Thus far, EMT in ovarian cancer cells exposed to malignant ascites was linked to the activity of the IL-6/IL-6R axis and signaling through the JAK2-STAT3 pathway [6]. The authors of another report postulated—agreeing with our observations—that EMT development in cancer cells isolated from malignant ascites is controlled by TGF-β1 signaling [9]. The significance of TGF-β1 is additionally strengthened by Smad 2/3 [21] and ILK [22], the transcription factors uniformly engaged in EMT in A2780 and SKOV-3 cells that are known to cooperate with TGF-β1. 

Interestingly, when the identified factors involved in ascites-induced EMT were neutralized, the procedure translated to the diminished invasion of cancer cells across monolayered PMCs, eventually confirming the pathophysiological relationship between these two phenomena. 

In conclusion, our mechanistic observations implied that the pathophysiology of the increased invasion of ovarian cancer cells toward the peritoneal stroma in the presence of malignant ascites is not limited to morphological alterations in PMCs [21] but also depends on dynamic changes (EMT) that occur in the malignant cells. This report also provides new targets for interventions aimed at preventing or limiting the intraperitoneal progression of ovarian cancer through the inhibition of ascites-dependent EMT. At the same time, further experiments are needed in which our findings will be verified using HGSOC. 

## 4. Materials and Methods 

### 4.1. Ascitic Fluids 

Malignant ascites were obtained at the time of cytoreductive surgery from chemotherapy-naïve patients with high-grade serous ovarian cancer (*n* = 8, 42–68 years of age). Benign fluids were collected from 8 patients (40–62 years of age) undergoing surgery due to the presence of *cystadenoma mucinosum multiloculare*, which is a benign cystic ovarian tumor. For experimental purposes, only 50 mL of both types of ascites were collected and processed. Upon collection, the fluids were centrifuged at 2500 rpm for 5 min, and then cell-free supernatants were stored at −20 °C. The malignant and benign ascites were assessed separately (not pooled) for the assays.

### 4.2. Cell Culture

The ovarian cancer cell lines A2780 and SKOV-3 were purchased from the ECCC (Porton Down, UK) and were propagated in RPMI 1640 medium (Sigma, St. Louis, MO, USA, cat. no. R0883) with a supplement (Sigma, cat no. G6784) containing l-glutamine (2 mM), penicillin (100 U/mL), and streptomycin (100 g/mL). The medium was enriched with 10% fetal bovine serum (FBS) (Life Technologies, Carlsbad, CA, USA, cat. no. 10270).

Human peritoneal mesothelial cells (PMCs) were isolated from pieces of omentum obtained from 8 patients undergoing elective abdominal surgery, as described elsewhere [23]. 

The cells were propagated in M199 medium (Sigma, cat. no. M2154) supplemented with l-glutamine (2 mM), penicillin (100 U/mL), streptomycin (100 µg/mL), hydrocortisone (0.4 µg/mL), and 10% FBS. 

### 4.3. Epithelial-Mesenchymal Transition (EMT)

Two markers of EMT were used in the study, E-cadherin (a marker of the epithelial phenotype) and vimentin (a marker of the mesenchymal phenotype). The incidence of EMT in cancer cells exposed to 10% malignant or benign ascites (for 7 days) was examined using immunofluorescence. After the incubation, the cells were fixed in paraformaldehyde (Sigma, cat. no. P6148), washed and treated overnight with antibodies against E-cadherin (Abcam, Cambridge, UK, cat. no. ab15148) diluted at 1:100 and antibodies against vimentin (Abcam, cat. no. ab16700) diluted at 1:100. The cells were extensively washed and incubated with DyLight 488 antibody (Abcam, cat. no. ab96899) diluted at 1:500 for 1 h at room temperature. Finally, the cells were washed, and the fluorescence emitted was recorded using a Synergy^TM^ 2 spectrofluorometer (BioTek Instruments, Winooski, VT, USA). 

Representative stainings of E-cadherin and vimentin were prepared using a PathScan EMT Duplex IF kit (Cell Signaling, Leiden, The Netherlands, cat. No. 7771). The immunoreactions were visualized using an Axio Vert.A1 microscope (Carl-Zeiss, Jena, Germany). 

In some experiments, the expression of E-cadherin and vimentin was quantified in the cancer cells exposed to the malignant and benign ascites, upon the fluid preincubation (for 72 h) with the neutralizing antibodies against EGF (R&D Systems, Abingdon, UK, cat. no. AF236, 30 ng/mL), HGF (R&D Systems, cat. no. MAB294, 1 µg/mL), IGF-1 (R&D Systems, cat. no. AF-291-NA, 200 ng/mL), and TGF-β1 (R&D Systems, cat. no. AF-101-NA, 400 ng/mL). In another group of experiments, the protein expression was evaluated in the cancer cells treated with the ascites, after preincubation (for 4 h) of the cells with the inhibitors of Smad 2/3 (SB 431542; Cayman Chemical Company, Ann Arbor, MI, USA, cat. no. CAS 301834-41-9, 200 nM), ILK (Cpd22; Calbiochem Merck, Darmstadt, Germany, cat. no. 407331, 1000 nM), AP-1 (3-aminobenzamide; Tocris, cat. no. 3544-24-9, 50 µM), and SP-1 (mithramycin A; Cayman Chemical Company, cat. no. CAS 18378-89-7, 10 nM). Specificity of the inhibitors has been confirmed in preliminary tests. The concentration of the antibodies and inhibitors, as well as the exposure times, were experimentally established in pilot studies. 

### 4.4. Invasion Assay

Analysis of transmesothelial cancer cell invasion was conducted using the Cultrex 96 Well BME Cell Invasion Assay (Trevigen Inc., Gaithersburg, MD, USA, city, country, cat. no. 3455-096-K). Briefly, PMCs (1 × 10^5^ cells per well) were seeded on the basement membrane extract (BME) to form a monolayer. Next, ovarian cancer cells exposed to 10% malignant and benign ascites (for 7 days) were placed into the upper chamber of the system (1 × 10^4^ cells per well), on top of the PMCs. The invasion of the cancer cells across the PMCs toward a chemotactic gradient generated by 1% FBS was monitored for 24 h. The intensity of fluorescence emitted by the cancer cells was recorded using a Synergy^TM^ 2 spectrofluorometer at 435 nm excitation and 535 nm emission wavelengths, respectively. 

In some experiments, invasion of the cancer cells through the PMCs was tested upon 72-h preincubation of the ascites with neutralizing antibodies against EGF, HGF, IGF-1, and TGF-β1. In another group of experiments, invasion was quantified using cancer cells treated with ascites, after preincubation (for 4 h) of the cells with the inhibitors of Smad 2/3, ILK, AP-1, and SP-1. In both groups of intervention studies, the concentrations of the antibodies and inhibitors were the same as described for the examination of EMT.

### 4.5. Statements

The study was approved by the Bioethics Committee at the Poznan University of Medical Sciences (consent numbers 187/14 and 543/14) and all patients gave their written informed consent. Moreover, we confirm that all methods were performed in accordance with the relevant guidelines and regulations. 

### 4.6. Statistics

Statistical analysis was performed using GraphPad Prism™ 7.00 software (GraphPad Software, San Diego, CA, USA). The means were compared using repeated measures analysis of variance (ANOVA) with the Newman-Keuls test as a post hoc test. When appropriate, the Wilcoxon matched pairs test was used. The results were expressed as the means ± SD. Differences with a *p* value < 0.05 were treated as statistically significant. 

## Abbreviations

EMT	Epithelial-Mesenchymal Transition
PMC	Peritoneal Mesothelial Cells
RFU	Relative Fluorescence Units
